# Clinical Report on Dysentery

**Published:** 1853-09

**Authors:** Austin Flint


### BUFFALO MEDICAL JOURNAL

AND

### MONTHLY REVIEW.

**VOL. 9.** SEPTEMBER, 1853. **NO. 4.**

### ORIGINAL COMMUNICATIONS.

ART. I.— *Clinical Report on Dysentery. Based on an analysis of forty-nine recorded cases.* By AUSTIN FLINT, M. D.

*" C'est en interrogeant fréquemment la nature que nous lui arrachons ses secrets."*

(Continued from page 157.)

### SECTION ELEVENTH.

*Description. Species. Definition. Name. Diagnosis.*

*Description.* Limited to the distinctive traits developed by the foregoing analysis, the following description will, in other words, be a synopsis of the more important of the facts which have been already seen to enter into the natural history of the disease.

Dysentery is not peculiar to any period of life, but attacks, by preference, the young and middle aged; it is not restricted to either sex, but it attacks a larger number of males than females.

It occurs both as a sporadic and epidemic disease; as a sporadic disease, it is generally mild, and very seldom fatal; as an epidemic, it is often a severe and fatal disease, presenting also certain semeiological and pathological characters not belonging to the sporadic form.

It occurs, in either form, during the months of July, August, September, and October, being very rarely observed in the remaining months of the year.

Persons apparently in good health, are as liable to be attacked by it as those who are feeble, or already suffering from different kinds of maladies.

In the great majority of cases the disease commences with simple diarrhœa, which continues one, two, three, or four days, and occasionally even longer, before the dysenteric characters make their appearance.

The distinctive symptoms pertain to the intestinal canal. The discharges contain mucus and blood in more or less abundance; the mucus sometimes in jelly-like masses, and sometimes in opaque flakes or laminæ. In severe cases a sero-sanguinolent fluid, more or less abundant, frequently accompanies the mucous discharges. Occasionally the dejections are observed to contain puruloid matter. The discharges generally are frequent, sometimes recurring after very short intervals; and usually, under these circumstances, they are quite small. They may contain, from time to time, fecal matter in greater or less quantity, except in very severe cases; and, in some instances, the fecal matter is in small, hard lumps, called scybala.

Associated with the dejections are tormina and tenesmus in most cases; but cases differ considerably as to the prominence of these symptoms, and in the cases attended with most danger, they are frequently the least prominent.

Abdominal tenderness is sometimes present, but rarely in a marked degree, and often absent. When present it may be found to be situated especially in some part of the tract of the large intestine.

Abdominal distension from meteorism is very rarely present, and, when present, slight.

Vomiting is an occasional symptom, and when present is an unfavorable event.

The tongue presents various appearances, but nothing distinctive of this disease.

Thirst is often present, and sometimes absent. The appetite is frequently lost, and in most cases more or less impaired.

The pulse, in mild cases, is but little accelerated. In severe cases, however, it becomes more or less frequent. The frequency being, ordinarily, in proportion to the danger of a fatal issue. Active symptomatic fever, in a small proportion of cases, accompanies the development of the disease; but, after the early stage, the pulse is generally either feeble, or vibratory, in proportion to the increased frequency. The increase in frequency and diminution in power, of the circulation, become more and more marked as the disease approaches a fatal termination. A sudden increase in the frequency of the pulse denotes an unfavorable change in the progress of the disease.

The mind, in mild cases, remains unaffected, and in the majority of fatal cases the intellect is preserved until death, or a short time previous; but some cases are characterized by delirium, which may be active, the patient even requiring restraint.

Sweating, and moisture of the surface, are events occasionally occurring both in fatal, and not fatal cases. They are not common. The skin, excepting the few instances in which symptomatic fever occurs, is cool, and in fatal cases usually the temperature is reduced in a notable degree, the reduction progressively increasing until the coldness becomes cadaveric.

No pulmonary symptoms are observable, or worthy of note, in the great majority of cases.

The duration of the disease varies from one day to twenty-one days, in the cases which recover, the average duration being about nine days; and in fatal cases, death occurs in from six to nineteen days, the average duration being also not far from nine days.

Relapses are not apt to occur, and convalescence is usually rapid.

The disease very seldom eventuates in chronic dysentery.

The mode of dying is by asthenia.

The fatality is limited to the epidemic form of the disease, and in this form varies considerably in different seasons.

In the great majority of cases, the disease cannot be traced to any obvious exciting causes.

When recovery takes place, the health is usually regained fully. The powers of the constitution are not permanently impaired, nor is there left a predisposition to any particular forms of disease. There are grounds for the opinion that persons who have once experienced the disease, are rendered less liable to be afterward attacked by it.

*Species.* The diversities under which dysentery is presented at different epochs, and, also, in different cases occurring at the same time and place, have led writers to institute several species of the disease. Thus, in some systematic works we find described species distinguished as typhous, bilious, intermittent and remittent, rheumatic, febrile and non-febrile, etc. Such subdivisions are of doubtful propriety, but it cannot be doubted that some of them are based on peculiarities which are interesting and important. The foregoing analysis of a limited number of cases, developed striking points of dissimilarity, viz., the sero-sanguinolent dejections in a certain proportion of cases; delirium in a small proportion, the mind maintaining its clearness in the remainder, and the presence or absence of well-marked symptomatic fever. These, and other variations, belong to the natural history of the disease, and, as such, claim the attention of the medical inquirer. But to consider them as giving rise to distinct species of the disease is, to say the least, an over-refinement of nosology, which complicates the subject without any corresponding advantages.

Some of the subdivisions of writers are based, not on variations in the phenomena of the disease, but on the coexistence of other diseases. Thus, dysentery may occur as a complication of the continued and the periodical fevers. Such are the cases, probably, that have been called cases of typhous, and remitting or intermitting dysentery. So rheumatism and dysentery are occasionally associated. These combinations are important in their practical relations, but it does not follow that the dysentery, under such circumstances, is specifically different.

The division into sporadic and epidemic dysentery is certainly appropriate. In its epidemic form, the disease may present symptoms and pathological characters rarely, if ever, observed in sporadic cases. Copious sero-sanguinolent discharges, for example, probably are peculiar to epidemic dysentery. Sporadic dysentery, as has been seen, is generally mild, and almost never fatal. The local affection, *i. e.*, the morbid changes within the intestine, have not the same extent, severity, and perhaps not the same character as those which are found, on dissection, in fatal cases of the epidemic form. Nevertheless, when the disease prevails as an epidemic, a certain proportion of cases are mild, and resemble, apparently, in all respects, those which occur sporadically, so that we might not be able, in individual instances, to declare that the disease belonged to the epidemic form. This fact renders it proper to distinguish between mild and severe cases of epidemic dysentery.

It is unnecessary to add, that the division into acute and chronic dysentery, is a valid one. The foregoing analysis, and the remarks appended thereto, have reference, as already stated, solely to acute dysentery.

*Definition.* Diseases are defined by selecting the most distinguishing traits from the symptoms exclusively, or by combining therewith facts derived from the morbid anatomy, together with, sometimes, an expression of the pathological character of the affection. In this instance, the symptoms pertaining to the intestinal discharges, furnish characters sufficiently precise and comprehensive for a definition which shall be free from hypothetical assumptions.

Dysentery is a disease, characterized by an abnormal frequency of evacuations from the bowels, containing mucus, with or without fibrinous flakes, usually mixed with blood, in some severe cases a discharge of thin sanguinolent fluid, puruloid matter occasionally being observed in the course of the disease, the evacuations accompanied by more or less tormina and tenesmus; occurring sporadically, and as an epidemic; cases of the former generally mild and almost uniformly ending favorably after a short career, but in the latter form frequently a grave disease, running a rapid course, ending fatally, by asthenia, in a ratio of cases varying at different epochs.

This comprises circumstances belonging to the natural history of the disease sufficient in number and character to distinguish it from other affections, and if so it secures all that is practically desirable in a definition.

*Name.* The disease is almost uniformly denominated by medical writers, as well as in common parlance, dysentery. The term does not derive any special significance from its etymology, but it has the negative merit of being free from error and hypothesis. Colonitis, or colitis, denote, what is true undoubtedly of a large proportion of cases, viz: the existence of inflammation in the colon; but it is not certain that in very mild cases the inflammation extends above the rectum. It is highly probable, indeed, that in some instances the local affection is confined to this part. Moreover, the disease in its epidemic, if not in its sporadic form, involves a morbid condition ulterior to the intestinal affection, so that a term limited in its signification to the latter does not comprehend all the disease. This criticism, however, might be applied to the names given to a large proportion of inflammatory affections; and the term dysentery is equally obnoxious to it.

Cases of the disease in which copious sanguinolent dejections take place have been called, popularly, and also by medical writers, *bloody flux.*

On the whole, with our present knowledge of the disease, it would be difficult to devise a title to be preferred to that which custom and long usage have assigned to it.

*Diagnosis.* The diagnosis of dysentery is not attended by any difficulties. The characters pertaining to the intestinal evacuations are so obvious and well-marked, that it would be difficult to confound it with any other affection. Cases of hemorrhage from the bowels are readily discriminated by the absence of mucus; and the discharge of pus from hepatic, or other abscesses opening into the alimentary canal, is neither preceded, nor accompanied by the symptoms invariably associated therewith in cases of dysentery.

### SECTION TWELFTH.

### *Treatment, and immediate (apparent) effects of remedies.*

In this section I shall complete the historical account of the cases analyzed, by giving the facts with respect to the treatment. I shall also offer any considerations respecting the effects of the remedies used, which an examination of these facts may suggest. It is obvious that in so small a number of observations the objects of study must be limited mainly to the immediate effects of remedies. A larger number of fatal and non-fatal cases is necessary for statistical data, by means of which the remote effects, *i. e.* the agency in producing death or recovery, of different measures of management, may be to some extent determined. I am careful to qualify the word effects in the caption of this section, by the term apparent. In estimating the immediate effects of remedies, a certain allowance is to be made for changes incident to the tendency of disease either toward recovery or death—changes due to the operations going on within the organism irrespective of medicinal influences. It is frequently very difficult to say how far an improvement in the symptoms, or the reverse, may be explicable in that way, and, hence, is a liability to err in judging of the immediate effects of remedies. This difficulty will diminish in proportion as the natural history and laws of disease are better established.

The remedies used in the treatment of the cases in this collection for the most part are divisible into the following classes: 1. Laxatives. 2. Mercury. 3. Opium. 4. Astringents, and topical applications by means of enemas. I will consider the facts pertaining to the use and immediate effects of these classes of remedies under distinct heads. I would remark here, that I do not propose in this section to discuss the subject of the management of dysentery. This I reserve for another place.

1. *Laxatives.* Laxatives were prescribed in only a small number of cases. The only laxative remedy used was castor-oil, exclusive of calomel, which, in a very few instances, was given in laxative, or cathartic doses; but these instances will be noticed under another head. In all the instances in which castor-oil was given, its operation was followed by the use of anodyne remedies administered by the mouth, or rectum. In no case did it constitute the chief measure of treatment, other remedies preceding, and as well as following it. In no instance does it appear to have been given more than once in the course of the disease. Judged by a comparison of the symptoms before its administration, and after its operation, the immediate apparent effects of the remedy, in eight cases, in which it entered into the treatment, were as follows: In two instances the affection appeared to be aggravated: that is, the dysenteric discharges denoted greater severity of disease after, than before the operation. In three instances the apparent effect was good, as shown by improvement in the dysenteric characters of the discharges. In the remaining three cases, no obvious change for the better, or worse, was observed.

In all the cases in which castor-oil was given, the disease ended in recovery.

I may remark that laxative remedies would probably have been used in a larger number of instances, and I should have tried those of the saline class, (which have been strongly recommended in this disease, of late years, by several writers,) had not most of the cases in this collection occurred in seasons when epidemic cholera prevailed to a greater or less extent. Under these circumstances, it was feared that the use of remedies of this class might be attended with a risk of inducing choleraic evacuations.

*Mercury.* Calomel was the only mercurial preparation given. This remedy was prescribed, more or less, in twenty-three cases. It was given, in some cases, in doses of one or two grains, repeated, usually, every four or six hours, but sometimes more frequently; in some cases in doses of five grains; and in other cases in doses still larger, viz: ten or fifteen grains. In almost every instance it was given in combination with some form of opium. In most of the cases, into the treatment of which calomel entered, other measures either preceded, or followed its use, or were pursued simultaneously. It is difficult, therefore, and indeed impossible, to isolate the operation of this, or any of the remedies sufficiently to determine with exact precision the effect, immediate or remote, which properly belonged to it in the cases severally. Some practical conclusions, however, may perhaps be reached, by comparing the apparent effect in different cases, and subsequently instituting comparisons between the present group of cases and those which were treated without mercury.

As the importance, or propriety of using mercury in dysentery is a subject which has occasioned considerable discussion of late, and respecting which there exists difference of opinion among practitioners, it may be more satisfactory to present a brief statement of the facts pertaining to this remedy contained in each of the histories distinctly. In doing this, as much conciseness as is practicable will be observed, in order not to weary the reader too much with tedious details. The duration of the disease, and the result, will be given in connection with each case.

No. 1. Calomel, gr. i, with Dover's powder, gr. ii, every four hours, and, at the same time, chalk mixture and paregoric, were prescribed on the fourth day. This was preceded by a dose of castor-oil. Patient was a child. Recovered. Duration of disease six or seven days.

No. 2. On the fourth day, calomel, gr. i, hourly, for six hours; then gr. $\frac{1}{2}$, every two hours, for two days, and an ounce of ung. hydrarg., in the mean time, applied endermically with friction. The patient was four years of age. No improvement of symptoms. Subsequently, calomel, grs. iii, every four hours, till free cathartic effect was produced, with bilious stools so called. No improvement. The remedy was then suspended, and the case treated with opium by the mouth and rectum, etc. Recovered. Duration not noted. This is one of the cases which persisted for some time in a chronic form.

No. 3. On the evening of the day on which dysenteric discharges appeared, 4 grs. of calomel were prescribed, and the remedy continued, in doses of grs. ii, every two hours, until 12 grains had been taken. The so-called bilious stools not being produced, another dose of grs. iv, was given. Copious green evacuations followed. To these succeeded profuse sero-sanguinolent discharges, and great prostration. The disease ended fatally on the third day after this treatment was pursued.

No. 4. At the commencement of the disease, calomel was given in doses of grs. ii, every four hours, with enemas of a solution of the sulphate of morphia and kreosote. Continued only for one day, and the sulphate of morphia substituted. On the seventh day, (symptoms becoming worse,) calomel again prescribed in doses of grs. iii, with opium grs. iss., every four hours. On the eighth and ninth days, improvement. On the tenth day (calomel continued) the discharges became sero-sanguinolent, the pulse rose rapidly in frequency, and the case ended fatally on the eleventh day.

No. 5. Calomel, grs. v, with opium, gr. ss., repeated twice, with four hours interval. Patient 8 years of age. Discharges diminished in frequency, and became more abundant, with but little blood and mucus, during following day and night. Calomel, grs. ii, with opium, gr. ss., every four hours, with enemas of laudanum, and semicupia, constituted the subsequent treatment. Recovered. Duration eight days.

No. 6. Calomel, grs. ii, with opium, gr. i, every four or six hours, was continued for three or four days. This treatment was commenced when ordinary diarrhœa only existed. The dysenteric discharges occurred under the treatment. The calomel was then suspended, and the case treated with acetate of lead and opium, tannin, morphia and quinia, with enemas of laudanum. Recovered. Duration five days.

No. 7. When dysenteric discharges appeared, calomel, in dose of about 10 grs., was given, and the remedy continued in doses of grs. ii, to grs. iii, combined with Dover's powder and opium, and laudanum enemas administered. The day but one after dysenteric discharges appeared, after an enema of a solution of morphia, gr. i, and of acetate of lead, gr. x, no dejection occurred for three days, and then a movement was obtained by an emollient enema. No looseness followed. Recovered. Duration, four or five days.

No. 8. Calomel, grs. xv, with opium, grs. ii, given shortly after the commencement of dysenteric discharges. No dejection till the following day, and then no mucus nor blood. The latter dejection followed by an enema of a decoction of oak bark, and no movement afterward for two days. Recovered. Duration, three or four days.

No. 9. Calomel, grs. x, to grs. xv, with opium, grs. ii. No dejection for twenty-four hours. On the following day, two or three evacuations occurred without dysenteric characters. Calomel, grs. v, and S. morphiæ, gr. $\frac{1}{4}$, with enema of laudanum. No dejection till the next day, when the bowels were moved by an enema. No recurrence of diarrhœa. Moderate ptyalism. Recovered. Duration, five or six days.

No. 10. Calomel in doses of grs. ii, every four or six hours, with opium. Dover's powder, the sulphate of morphia was continued for several days. Afterward anodynes by mouth and rectum, acetate of lead, and tannic acid. Fatal on the tenth day.

No. 11. The dysenteric discharges appeared, in this case, while the patient was taking calomel in small doses. When the dysenteric discharges appeared, calomel, grs. v, with opium, grs. ii, was given. In a short time it was repeated, with T. opii, gtt. 100, pain being severe. On the following day, ptyalism was observed, and became severe. Subsequent treatment consisted of morphia, acetate of lead, tannic acid, diffusible stimulus. As respects the several symptoms, this was, perhaps, the gravest of the cases ending in recovery. Duration, eight days.

No. 12. Treated with opium till the third day, when there was an increased severity of the symptoms. Then calomel prescribed in doses of grs. v, with opium, grs. ii, every four or six hours. Afterward had but two dejections in twenty-four hours, and speedily convalesced. Duration, five days.

No. 13. On the second day, calomel, grs. iii, with opium, grs. ii, every four or six hours. No improvement on the third day, when the calomel was suspended, and the opium continued. On the fourth day, symptoms improved. On the fifth, dejections became more frequent, with more blood and mucus, and the calomel was resumed in doses of grs. iii, with opium, grs. ii, every four or six hours. On the sixth day, no improvement, and castor-oil was given, followed by opium and astringents. Under this treatment recovery took place. Duration, eight days.

No. 14. First day after admission into hospital, the treatment consisted of T. opii, gtt. xxx, every six hours, and enemas of laudanum. Second day, symptoms improved. Calomel, grs. x, with opium, gr. i, prescribed on this day. Third day marked improvement. Opium continued. Recovered. Duration, not determinable.

No. 15. Symptoms improved under anodyne remedies, but on the ninth day the discharges became more frequent and bloody. On the tenth day, calomel, grs. xv, with opium, grs. iii, was given. Discharges through this day less frequent and bloody. Opium in dose of grs. iii, given at night; and continued the next day. Speedy convalescence from this date. Precise duration not determinable.

No. 16. On first day after admission into hospital, sulphate of morphia, gr. $\frac{1}{4}$, and sulphate of quinia, grs. ii, every six hours, with enemas of laudanum. Symptoms relieved, but on second day, calomel in dose of grs. x, was given, without opium, an enema of a strong solution of the nitrate of silver being added. On the third day improvement; dejections not frequent and free from mucus. The same dose of calomel, and the same injections were repeated. Fourth day, symptoms about the same. S. morphiæ, gr. $\frac{1}{4}$, and S. quiniæ, grs. ii, every six hours. Speedy convalescence. Duration, eight days.

No. 17. On admission, fifth day after attack, calomel, grs. xv, with opium, grs. iii, was given. No dejection in day time, but several during the following night. Sixth day, calomel and opium repeated in the morning, and at night opium, grs. iv, given. No dejections till midnight. After midnight, eight or ten. Third day, dejections devoid of blood and mucus. Subsequent treatment consisted of T. opii, T. kino, nitrate of silver, and sulphate of quinia. Recovered. Duration, eleven days.

No. 18. Admitted eighth day of disease. Several doses of calomel, grs. v, with opium, gr. i, given on the first day, and enema of laudanum. On second day, sulphate of morphia, sulphate of quinia, acetate of lead and brandy. Fatal on ninth or tenth day.

No. 19. Admitted five days after attack (having had no previous treatment.) The treatment consisted of the sulphate of morphia, by mouth and enema, till the fourth day after admission, when calomel, grs. ii, with Dover's powder, grs. iv, every four hours, was prescribed. On the following day the discharges were stercoraceous, large, and without blood. The day previous they had contained blood. Calomel omitted, sulphate of quinia given by the mouth, and sulphate of morphia by enema. Recovered. Duration, sixteen days.

No. 20. On admission, two days after the attack, calomel, grs. v, with Dover's powder, grs. v, was given every six hours. Ptyalism was produced. Recovered. Duration, fourteen days.

No. 21. Calomel in small doses, with opium and injections of laudanum and tannic acid constituted the sole measure of treatment. Recovered. Duration, fourteen days.

No. 22. Calomel in small doses, with opium and injections of laudanum and tannic acid, constituted the sole measures of treatment. Recovered. Duration not noted.

No. 23. Calomel in small doses, with opium, and injections of laudanum, constituted the treatment. Ptyalism was induced. Under this treatment the dejections became sero-sanguinolent, and death occurred on the seventh day.

It will be seen, on examination of the facts pertaining to the use of calomel contained in the foregoing abstracts, that in three instances only was this remedy given uncombined with opium. (Nos. 2, 3, and 16.) In one of these cases (No. 2) the effect, so far as could be judged by the subsequent symptoms, was not favorable; in another, (No. 3) it appeared to be unfavorable, and in the remaining instance, (No. 16) the dose in this case being larger, (grs. x, given on two successive days,) the effect was apparently good. It is obviously impossible to draw any decided inferences from the apparent results in these few instances.

In the remaining twenty cases, the calomel was invariably combined with opium. This being so, it is far easier to indulge opinions as to the amount of efficacy belonging to the combination, or to each remedy separately, than to establish their correctness. In four of the cases (Nos. 7, 8, 9, 12) the apparently good effect was striking. In six cases (Nos. 5, 14, 15, 16, 17, 19) the effect, so far as could be judged by the symptoms before and afterward, was good.

In one case only (No. 3) does such a comparison of the symptoms lead to a very strong suspicion of a positively bad effect.

In the remainder of the cases, either the apparent effect was negative, or indeterminate.

It is worthy of notice that in two cases (Nos. 6, 11) dysenteric discharges occurred while the patients were taking calomel which had been prescribed for the preliminary diarrhœa.

It is also to be noticed that, of the twenty-three cases in which calomel was used, five were fatal cases. This leaves six fatal cases for the remaining twenty-six cases; so that the ratio of fatality is very nearly equal in the two groups of cases distinguished from each other by the use and non-use of calomel.

What conclusions respecting the value of calomel in dysentery shall be deduced from these data? We may be better prepared to answer this question after examining the facts in the cases, into the treatment of which the use of calomel did not enter. At present, as it seems to me, we are authorized to say that the evidence of any positively pernicious effects from the use of calomel, so far as concerns the symptoms and issue of the disease, is quite small; and, on the other hand, the circumstances connected with its use in several of the cases would seem to afford support to the conjecture that it may have exerted a salutary influence.

*Opium.* Under the head of opium I include the use of the salts of morphia, and the pulvis opii et ipecacuanhæ. Opiates were given by the mouth, and by enema. Generally both methods of administration were conjoined. In no case in the whole collection were anodyne remedies entirely excluded from the treatment. Of the cases in which calomel was not used, in several the medicinal treatment consisted wholly of remedies of the class under consideration; but in other cases, the use of astringents, etc., were conjoined. In the cases last referred to, however, the main reliance was on the opium with which the astringents were almost always combined.

In order that the reader may have before him the facts contained in the cases in which opium was used without calomel, and be better able to compare these facts with those already presented in the cases in which calomel was given, I will adopt the course which was pursued under the head of *mercury* and proceed to give an abstract of the facts pertaining to the use of opium in the cases severally, from the treatment of which calomel was excluded.

No. 1. First day, Dover's powder. Second day, castor-oil. Third day, opium, gr. i, and in afternoon chalk mixture and paregoric. Distinct amelioration of symptoms in evening. Chalk mixture and paregoric the only remedy afterward given. Recovered. Duration, five days.

No. 2. Treated with Dover's powder and opium, and laudanum injections. Symptoms relieved. Patient passed into other hands, and duration not ascertained. Recovered.

No. 3. Laudanum enemas constituted the sole treatment. The relief was immediate. Recovered. Duration, four days.

No. 4. Opium by the mouth, and laudanum enemas, constituted the sole treatment. Laxative required after convalescence, owing to constipation. Recovered. Duration, ten days.

No. 5. Came under observation on the seventh day. Opium and the acetate of lead, tannic acid with laudanum enemas, stimulants, and sinapisms, constituted the treatment. Fatal on ninth day.

No. 6. Treatment consisted of opium, Dover's powder, morphia,* and the acetate of lead by the mouth; laudanum injections; wine whey. Recovered. Duration, thirteen days.

No. 7. Opium and Dover's powder, with laudanum enemas. The latter produced marked relief. Recovered. Duration, eight days.

---

* For brevity, the terms morphia and quinia will be used. The salt of morphia usually employed was the sulphate.

No. 8. Came under treatment sixth day. Had remedies, previously, from a German empiric. No relief obtained. Immediate relief followed the use of opium, gr. i, every four hours. This the only treatment. Recovered. Duration, ten days.

No. 9. Laudanum enemas constituted the sole treatment. These produced prompt and marked relief. Recovered. Duration, seven days.

No. 10. T. opii camph. by mouth, and laudanum enemas. Symptoms promptly relieved by these measures. No other treatment. Recovered. Duration, seven days.

No. 11. Opium in small doses, Dover's powder, morphia, and the acetate of lead,* constituted the treatment. Recovered. Duration, ten days.

No. 12. Morphia, gr. $\frac{1}{4}$, with quinia, gr. ii, every four hours, constituted the treatment for four days. Then, opium, grs. ii, every four hours, and morphia, grs. ss., by enema. This was the sole treatment. Recovered. Duration not determinable.

No. 13. Admitted into hospital a week after date of attack. Morphia, gr. $\frac{1}{4}$, every six hours. This was the sole treatment, except quinia, grs. ii, three times the day before his discharge. Recovered. Duration, twelve days.†

No. 14. Admitted on fourth day of disease. First day, morphia, gr. $\frac{1}{4}$, every four hours, and gr. i, as suppository. Second day, morphia, gr. ss., acet. plumbi, grs. iv, every six hours, with enema of nit. argenti, grs. x, aquæ ℥i., followed by enema of T. opii, ʒii. Third day, morphia, gr. ss., every six hours. Fourth day, morphia continued, with enemas of morphia and the acetate of lead. Fifth day, treatment continued. Sixth day, improvement. Morphia, gr. $\frac{1}{4}$, and quinia, grs. ii, every six hours. Seventh day, dysenteric discharges ceased. Treatment continued. Subsequently some return of

* In making the preliminary analysis, whence these abstracts are taken, the doses of the remedies are frequently not stated, nor the immediate apparent effects. This is the explanation of the omissions on these points which will be noticed. It would require great labor to supply the omissions, and the object is not deemed of sufficient importance.

† The duration here, and subsequently, dates from the attack, not from the time of admission into hospital.

dysenteric discharges, and morphia, gr. ss., every six hours, was resumed. A dose of castor-oil was given, followed by opium, grs. iii. Recovered. Duration, nine days.

No. 15. Admitted a week after date of the attack. Morphia, gr. ¼, with quinia, grs. ii, continued regularly for a week; and, with laudanum enemas, constituted the sole treatment. Recovered. Duration, fourteen days.

No. 16. Admitted third day of disease. Morphia, gr. ¼, and quinia, grs. ii, every six hours, with laudanum enemas. Fourth day of disease, improvement. Morphia, gr. ss., morning and evening, and enemas of morphia, gr. i, and acetate of lead, grs. x. Fifth day, dejections thin, feculant, and free from blood and mucus. Treatment continued. Progressive improvement. Castor-oil given on the ninth day, and its operation followed by opium, grs. iii. Recovered. Duration, sixteen days.

No. 17. Admitted third day of disease. Morphia, gr. ss., morning and evening, with enemas of morphia, gr. i, and acet. plumb., grs. x. This treatment continued till convalescence. Recovered. Duration, ten days.

No. 18. Admitted fourth day of disease. Had received treatment, nature of which was unknown. Castor-oil, and after operation, morphia, gr. ss., morning and evening, with enemas of morphia, gr. i. Second day, treatment continued. Third day, morphia, gr. ¼, with quinia, grs. ii. Continued till convalescence. Recovered. Duration, twelve days.

No. 19. Admitted seventh day of disease. Dover's powder, grs. x, with laudanum enemas, and suppository of morphia, gr. i. Second day, Dover's powder, grs. x, and suppository of morphia, gr. i. Third day, castor-oil, and after operation, Dover's powder, grs. x, and suppository of morphia. Fourth day, marked improvement. Morphia, gr. ¼, with quinia, grs. iii, every six hours, and enema of nitrate of silver, grs. xx, in an ounce of water. Fifth day. Continued morphia and quinia, and repeat enema. The morphia and quinia continued until convalescence. Recovered. Duration, twenty-one days.

No. 20. First day after admission, opium, gr. i, every four hours, and enema of morphia, gr. i. Second day, opium, gr. i, every three hours, and enema of nitrate of silver, grs. x, in two ounces of water. Third day, morphia, gr. ¼, acetate of lead, grs. ii, every four hours. Carb. of ammonia and brandy. Fourth day, opium, gr. iss., every four hours, with enema of morphia and tannic acid. Brandy and carb. ammoniæ. Fifth day, opium, grs. ii, acetate of lead, grs. iv, camphor, grs. iii, every six hours. Enema of nitrate of silver, gr. i, in two ounces of water. Fatal on the eighth day after admission.

No. 21. Admitted second day of disease. First day, castor-oil, and operation followed by opium, gr. i, every four hours, and laudanum enema. No evacuation after enema for entire night. Second day, discharges of blood and mucus returned. Opium, gr. i, every four hours, and enemas of morphia, gr. i. Third day, morphia, gr. $\frac{1}{4}$, every four hours. Fourth day, discharges natural. Recovered. Duration, seven days.

No. 22. Admitted second day of disease. Treatment consisted of opium, gr. i, to grs. ii, every three hours, and laudanum enemas. Fatal on the sixth day.

No. 23. Morphia, gr. $\frac{1}{4}$, every four hours, and laudanum enemas. Third day, opium, gr. ss., and acetate of lead, grs. ii, every two hours. Fourth day, morphia, gr. ss., hourly, for several hours. Brandy. Fatal on the eighth day.

No. 24. Patient aged 11 years. Morphia, gr. $\frac{1}{8}$, and laudanum enema. Discharges arrested immediately. Recovered. Duration, four days.

No. 25. Treatment consisted at first of morphia, gr. ss., every four or six hours, and laudanum enemas. Second day, opium, gr. ss., and acetate of lead, grs. ii, hourly. Third day, the same. Fourth day, opium, gr. i, hourly, with brandy. Fifth day, opium, gr. i, every alternate hour, and grs. ii, every alternate hour. Sixth day, opium, grs. ii, hourly. This was continued for several days, with marked improvement as respects frequency of the dejections, but the sero-sanguinolent character which they had at first was preserved. Tannic acid and matico given in this case. Fatal on the seventeenth day.

No. 26. Treatment consisted in the use of morphia, and sometimes, conjoined, the acetate of lead. Stimulants were given freely. Dejections sero-sanguinolent from an early period in the disease. Morphia given in doses of from one to two grains every alternate hour, without inducing narcotism, the symptoms becoming aggravated on suspending the use of morphia. Fatal on the ninth day.

An examination of the facts, as thus presented, in this group of cases, with reference to the immediate apparent effect of the use of opium without calomel, leads to the following results: The effect appeared to be strikingly good in four cases (Nos. 3, 7, 8, 24) which it will be observed, is precisely the same number as in the former group.

It appeared to be decidedly good, but in a less striking degree, in nine cases (Nos. 1, 2, 4, 9, 10, 14, 15, 16, 17.) The number of cases in which this effect is apparent in the present group exceeding that in the former group by three.

The effect is indeterminate, owing to the conjoined use of other measures, and, in some instances, defect in making the analysis, in the remainder of the cases, exclusive of those which were fatal.

No evidence appears of an unfavorable effect in any case.

Six of the cases in this group were fatal. In some of these cases, it may be remarked, the immediate apparent effect of the use of opium was good, as shown by a marked aggravation of the symptoms when the remedy was temporarily suspended. This was true especially in the cases numbered 25 and 26.

The inquiry may arise, how do the cases, in the two groups, in which a striking immediate effect was apparent, in the one group from calomel and opium, and in the other group from opium alone, compare as respects the quantity of opium given. If the reader will take pains to refer to the four cases alluded to in each group, (viz: in the first group Nos. 7, 8, 9, 12, and in the second group Nos, 3, 7, 8, 24) he will find that more opium was given with the calomel in the cases belonging to the first group, than was given alone in those belonging to the second group!

On comparison, also, of the cases in the two groups in which a good effect was apparent, but less striking in degree, it will be found that the quantity of opium given in both is not very far from equal.

A perfectly fair comparison of the results of treatment in the two groups, requires that the cases should be as equal, in all respects, as possible. The two groups should embrace about the same number of mild, and severe cases. The cases occurring at different seasons should be equally divided, since we have seen that the tendency to a fatal result is greater in some seasons than in others. About an equal number, also, of hospital cases, and those in private practice, should belong to both. In determining the facts as far as practicable with reference to these points, it will suffice to limit the examination to those cases, in both groups, in which the immediate effect of the treatment appeared either strikingly, or decidedly good. These cases, in the first group, *i. e.*, the group of cases treated with calomel and opium, are ten in number. In the second group, *i. e.*, the cases treated without calomel, they are thirteen in number.

So far as judgment can be formed by a general survey of the histories, the two lists embrace about an equal relative proportion of mild and severe cases.

Of the ten cases in the first group, five were hospital cases, and five occurred in private practice. Of the thirteen cases in the second group, four were hospital cases, and nine occurred in private practice.*

Of the ten cases in the first group, all the ten occurred in 1849. Of the thirteen cases in the second group, four occurred in 1849, four in 1848, two in 1846, and in 1838, 1847, and 1851, in each year one case. In explanation of the difference in the two groups as respects the years, it should be stated that in 1849 I prescribed calomel and opium in most of the cases coming under observation, and in but a small portion of cases before, and after that year.

Directing attention to the fatal cases among those treated with calomel and opium, and those treated with opium without calomel; of the five fatal cases in the first group, one was a hospital case, and the remaining four were in private practice. Of the six fatal cases in the second group, two were hospital cases, and four occurred in private practice. In this particular they are as equal as possible.

Of the five fatal cases in the first group, four were in 1849, and one in 1850. Of the six fatal cases in the second group, one was in 1848, two in 1850, one in 1851, and two in 1852.

Of the five fatal cases in the first group, one occurred in July, three in August, and one in September. Of the six fatal cases in the second group, one occurred in July, two in August, and three in September.

Repeating now an inquiry before made, 'What conclusions respecting the value of calomel in dysentery shall be deduced from these data,' a review of all the facts seems to warrant the belief that, so far as these observations furnish evidence on the point, it is adverse to the opinion of any favorable influence being exerted by this remedy on the morbid processes involved in the disease. It may be said, and with justice, that the number of observations is

* Of the whole twenty-three cases treated with calomel and opium, nine were hospital cases, and fourteen occurred in private practice; and of the whole twenty-six cases treated without calomel, eleven were hospital cases, and fourteen occurred in private practice. So that in the distribution of the cases as respects this point, the two groups are about as equal as possible.

insufficient to settle positively this practical question; but admitting this, are the notions on the subject more likely to be nearer the truth which are formed without any interrogation of recorded cases?

If the conclusion we have deduced respecting the value of calomel be correct, it follows, assuming the immediate apparent effect of the calomel and opium in the first group of cases not to have occurred irrespective of therapeutic influences, that the opium given in combination with the calomel was the efficient agent. These cases, therefore, as well as those in the second group, go to show the efficacy of opium in the management of dysentery. It would certainly be more satisfactory, in a scientific point of view, if we were able to compare the results which we have presented, with the facts contained in an equal number of cases which were treated without opium; but there is not much prospect of such a collection of cases being placed at the disposal of the analyst.

There still remains a point of comparison between the cases treated with calomel and opium, and those treated with opium alone, which may have some interest. This is the relative duration of the disease in these two groups. In examining with reference to this point, it will be proper to divide the cases in each group into (1) those treated in hospital, and (2) those treated in private practice. Making this division the results are as follows:

| | |
|---|---|
| 1*st. Mean duration of disease in four hospital cases treated with calomel and opium:—* | *Do. in eight hospital cases treated without calomel:—* |
| $13\frac{1}{2}$ days. | $13\frac{7}{8}$ days. |

The similarity in these results is interesting.

| | |
|---|---|
| 2*d. Duration in eight cases in private practice treated with calomel and opium:—* | *Do. in nine cases in private practice treated without calomel:—* |
| $6\frac{1}{8}$ days. | 7 6-9 days. |

The duration, it is observed, is somewhat greater in the cases treated without calomel. It may be a question whether this slightly greater duration be owing to difference of treatment, or to a proportionately greater severity of disease.

The fatal cases are not included in the above.

| | |
|---|---|
| 3d. *Duration in five fatal cases treated with calomel and opium:—* | *Do. in five fatal cases treated without calomel:—* |
| 10 4-5 days. | 10 2-5 days. |

Here, again, the coincidence developed by the comparison is striking.

*Astringents, and topical applications by means of enemas.* The astringent remedies used were the acetate of lead, tannic acid, the extract of rhatany, the nitrate of silver, and the tincture of kino. They were used in a small proportion of the cases only, and always in conjunction with opium, or the salts of morphia, with or without the addition of calomel. The difficulty attending the task of endeavoring to determine by an analysis of the histories whether any immediate effects could be attributed to these several remedies in the different cases, and the small probability of arriving at results sufficient to repay the labor, lead me to content myself with stating the fact simply that these remedies entered into the treatment to a limited extent.

*Topical applications by means of injections* were used in several cases. The tannic acid, in mixture, was sometimes given in that way, always in conjunction with laudanum. The apparent effect, occasionally, was to render the opiate more likely to be retained. This was the object for which it was given.

The acetate of lead, in solution, was given repeatedly, always conjoined with laudanum, or the acetate of morphia, the object being to obtain a local astringent operation. What value belongs to this remedy, thus used, does not appear in the histories.

A strong solution of the nitrate of silver was given, by enema, in a small number of instances. The facts pertaining to this measure are briefly as follows:

No. 1. Nitrate of silver, grs. x, dissolved in two ounces of water, was given without any marked effect. The case ended fatally.

No. 2. Nitrate of silver, grs. xx, in an ounce of water, was given; it occasioned considerable pain, and no other apparent effect. It was subsequently repeated, and again occasioned severe pain, without any appreciable influence being produced on the disease.

No. 3. Nitrate of silver, grs. x, in an ounce of water, was given. It occasioned pain, and was shortly followed by a laudanum enema.

No. 4. Nitrate of silver, grs. x, in an ounce of water, was given at 5, P. M. and repeated at 9, P. M. It does not appear to have occasioned pain. The next day there was marked improvement, and convalescence speedily occurred.

No. 5. Nitrate of silver, grs. xx, in two ounces of water, was given, and, at the same time, calomel, grs. x, uncombined with opium, or any other remedy. The following day there was a marked improvement in the symptoms. It does not appear to have occasioned pain in this instance.

No. 6. Nitrate of silver, grs. x, in an ounce of water, was given in this case, and repeated twice daily, for several days. The patient was a child four years of age. They appeared to occasion marked relief; no pain followed, and the frequency of the dejections was apparently diminished by them.

The solution of the nitrate of silver was administered by means of a small glass syringe, and could not have been projected above the lower part of the rectum. Under these circumstances it is obvious that the medicine can only be of service in relieving symptoms dependent on the morbid condition in that part of the intestine, the topical application extending only over a small portion of the mucous surface affected, generally, in this disease.

Injections of kreosote, combined with mucilage, were administered in a few instances, the object being to relieve the tenesmus, and favor the retention of anodyne enemas. Their success does not appear from the histories.

Injections of laudanum, or a solution of the sulphate of morphia, belong under the head of opium. They were found more or less useful in almost every case, and in some instances, the apparent effect was very marked. A few cases were treated by these injections exclusively. Suppositories were occasionally used. The advantage of injections was often lost by their being quickly expelled. Inability of retaining them was a difficulty which generally followed, after a time, their frequent repetition. Under these circumstances suppositories sometimes were more successful.

The warm hip bath was found very serviceable in a few cases, the patients being children. The local symptoms were relieved in a striking manner by this measure. Particularly in one case, (the patient four years of age,) in which the disease persisted for some time, the hip-bath produced a soothing effect and suspended for a time the frequent evacuations, after enemas had ceased to be retained.

Finally, tonic remedies, and stimulants, were employed more or less. The sulphate of quinia was given in a number of cases, in combination with opium, or the sulphate of morphia. Diffusible stimulants were given whenever the symptoms denoted failure of the vital forces, as shown particularly by feebleness and frequency of the pulse. Brandy was the form of stimulant generally used, and the quantity given was in proportion to the indications of a tendency to a fatal issue by asthenia.

[A Supplement to this Report will appear in the next number.]

